# TRANSLATION, CULTURAL ADAPTATION, AND SEMANTIC VALIDATION OF THE PEDIATRIC NEUROGENIC BOWEL DYSFUNCTION SCORE (NDBS) INTO BRAZILIAN PORTUGUESE AND A PILOT STUDY

**DOI:** 10.1590/S0004-2803.24612025-034

**Published:** 2025-09-05

**Authors:** Lenamaris Mendes Rocha DUARTE, Ana Lúcia Ribeiro SALOMON, Carmelia Matos Santiago REIS

**Affiliations:** 1 Rede SARAH de Hospitais de Reabilitação, Departamento de Pediatria, Brasília, DF, Brasil.; 2 Escola Superior de Ciências da Saúde, Fundação de Ensino e Pesquisa em Ciências da Saúde, Brasília, DF, Brasil.

**Keywords:** Translating, cultural competency, validation studies, spina bifida, neurogenic bowel, pediatric, quality of life, Tradução, competência cultural, estudos de validação, espinha bífida, disfunção intestinal neurogênica, pediatria, qualidade de vida

## Abstract

**Objectives:**

This study aimed to translate the Neurogenic Bowel Dysfunction Score into Brazilian Portuguese, adapting it culturally and validating it semantically.

**Methods:**

The process followed international guidelines for translation, back-translation, cultural adaptation, and semantic validation, involving a committee of specialists and a pre-test with 10 Brazilian pediatric patients with neurogenic bowel dysfunction (mean age: 11 years). Participants were divided into two groups, depending on whether they used transanal irrigation for intestinal management. The translated version was evaluated considering its clarity, equivalence (Likert scale), Kendall’s Coefficient of Concordance, and applicability.

**Results:**

The Brazilian version of the pediatric Neurogenic Bowel Dysfunction Score, presented here, showed high levels of linguistic and cultural equivalence (Kendall greater than 0.8) according to the specialists, after the second round of evaluations. Furthermore, participants understood the questionnaire very well (mean clarity on a Likert scale: 4.7±0.1). The groups were homogeneous for most variables analyzed. The score of Group 1, which used transanal irrigation, was found to be less severe than that of Group 2 (*P*=0.004). Group 1 showed more satisfaction with their bowel function control than Group 2 (*P*=0.008).

**Conclusion:**

The initial validation of the pediatric Neurogenic Bowel Dysfunction Score is a step forward in its integration into the national clinical context. The instrument was found to be reliable and viable for use in clinical practice and research, enabling standardized assessments and global comparisons. Its implementation will help ensure efficient neurogenic bowel dysfunction management and improve the health and quality of life of these children and adolescents.

## HIGHLIGHTS

• This study aimed to translate the Neurogenic Bowel Dysfunction Score into Brazilian Portuguese.

• It followed international guidelines for translation, back-translation, cultural adaptation, and semantic validation, involving a committee of specialists and a pre-test.

• The Brazilian version demonstrated high levels of linguistic and cultural equivalence according to specialists, and participants understood the questionnaire very well. Group 1, which used transanal irrigation, had less severe neurogenic bowel dysfunction and reported greater satisfaction with their bowel function control.

• The instrument was found to be reliable and feasible for use in clinical practice for the neurogenic bowel dysfunction management and research, allowing for standardized assessments and global comparisons.

## RESUMO

**Objetivos:**

Este estudo teve como objetivo traduzir o *Neurogenic Bowel Dysfunction Score* para o português brasileiro, adaptá-lo culturalmente e validá-lo semanticamente.

**Métodos:**

O processo seguiu diretrizes internacionais para tradução, retrotradução, adaptação cultural e validação semântica, envolvendo um comitê de especialistas e um pré-teste em 10 pacientes pediátricos brasileiros com disfunção intestinal neurogênica (idade média: 11 anos). Os participantes foram divididos em dois grupos, dependendo do uso da irrigação transanal para o manejo da disfunção intestinal. A versão traduzida foi avaliada quanto à sua clareza, equivalência (escala de Likert), coeficiente de concordância de Kendall e aplicabilidade.

**Resultados:**

A versão brasileira do *Neurogenic Bowel Dysfunction Score* pediátrico, apresentada neste estudo, demonstrou altos níveis de equivalência linguística e cultural (coeficiente de Kendall superior a 0,8) de acordo com os especialistas, após a segunda rodada de avaliações. Além disso, os participantes compreenderam muito bem o questionário (média de clareza na escala de Likert: 4,7±0,1). Os grupos foram homogêneos para a maioria das variáveis analisadas. A disfunção do Grupo 1, que utilizou irrigação transanal, foi considerada menos grave do que a do Grupo 2 (*P*=0,004). O Grupo 1 demonstrou maior satisfação com o controle de sua função intestinal se comparado ao Grupo 2 (*P*=0,008).

**Conclusão:**

A validação inicial do *Neurogenic Bowel Dysfunction Score* pediátrico representa um avanço para sua integração no contexto clínico nacional. O instrumento mostrou-se confiável e viável para uso na prática clínica e na pesquisa, possibilitando avaliações padronizadas e comparações globais. Sua implementação contribuirá para um manejo mais eficiente da disfunção intestinal neurogênica e para a melhoria da saúde e qualidade de vida dessas crianças e adolescentes.

## INTRODUCTION

Neurogenic bowel dysfunction (NBD) is a condition characterized by the loss of neuromuscular control over one’s intestinal tract, due to interruptions in the communication between the central nervous system and the bowel^1^ ^,^ ^2^. Affecting more than 80% of individuals with spina bifida (SB), NBD manifests in a wide range of symptoms, including constipation, incomplete bowel movements, fecal incontinence, and complications such as abdominal pain, hemorrhoids, anal fissures, anorectal bleeding and prolapse, fecalomas, and colonic dilation^2^ ^-^ ^6^. In addition to its physical repercussions, the NBD compromises psychosocial aspects, having a negative impact on one’s self-esteem, daily activities, and school performance, while also being a negative influence on the social integration of these individuals^7^ ^,^ ^8^.

NBD management is a major challenge, requiring a multidisciplinary approach with conservative strategies such as educating caregivers, providing dieting orientation, adequate water intake, abdominal massages, toilet training, perianal stimulation, and manual extraction of feces. Pharmacological therapies such as oral laxatives, suppositories, fleet enemas, as well as bowel emptying techniques, such as transanal irrigation (TAI) with warm water, are also widely used^1^ ^-^ ^3^ ^,^ ^9^. Biofeedback-based interventions must have their efficiency proven in the long term^5^ ^,^ ^6^ ^,^ ^10^, while innovative therapies, such as sacral neurostimulation, are increasingly common in research on the field, despite their high cost and limited access^11^. In resistant cases, surgical intervention may be an option, as long as it is carefully evaluated, considering the risks, benefits, and impacts on one’s quality of life^12^ ^,^ ^13^.

Standardized instruments, such as the Neurogenic Bowel Dysfunction Score (NBDS), developed by Krogh et al., 2006^14^, as well as its pediatric version, adapted by Kelly et al., 2016^15^, are widely recognized as reliable methods to measure the severity of NBD, orient interventions, and monitor therapeutic responses^14^ ^-^ ^16^.

The original NBDS^14^ has been translated and validated in several languages, such as Spanish^17^, Arabic^18^, Dutch^19^ and German^20^, including cultural adaptations to ensure its local relevance and the validity of its results, as well as comparisons between different cultures. Nevertheless, the NBDS^15^ is yet to be translated into Brazilian Portuguese or validated in that language, limiting its applicability in the Brazilian context.

In Brazil, despite the existence of NBD management programs in specialized centers, the access to specialized multidisciplinary care is limited and out of the reach of many patients and their families^21^ ^,^ ^22^. There are no necessary resources to implement individual therapeutic plans such as assistance devices, educational support, and longitudinal follow-up, which worsens clinical complications and increases psychosocial impact. This shortcoming shows how necessary it is to validate instruments in the country’s language, as this can help health workers perform standardized assessments of this condition and plan individualized strategies^1^ ^,^ ^2^ ^,^ ^5^ ^,^ ^16^ ^,^ ^23^.

This study aims to translate the pediatric NBDS^15^ into Brazilian Portuguese, adapt it culturally, and carry out a preliminary validation of the resulting instrument. The implementation of this survey aims to improve NBD clinical management, providing support to evidence-based therapeutic decisions, promoting the social integration of children and adolescents with SB, and helping the elaboration of public policies to improve the quality of life of this population.

## METHODS

### Authorization and ethical compliance

The study started after the author of the original pediatric NBDS, Maryellen S. Kelly^15^, gave her authorization for the translation of this survey into Brazilian Portuguese, including a cultural adaptation and validation process.

The research followed standardized international guidelines for translation, back-translation, cultural adaptation, and semantic validation^24^. This study was approved by the Research Ethics Committee of the SARAH Network of Rehabilitation Hospitals (Opinion No.: 6,565,133) and complied with the ethical principles established by the National Health Council in Resolution No. 466/2012.

### Assessment instrument

The pediatric NBDS, developed by Kelly et al. in 2016^15^, was selected due to its ability to measure NBD symptoms and their impact on the health and quality of life of children and adolescents from 6 to 18 years old, with SB.

The questionnaire includes 15 questions, with a total score that can vary from 0 to 41. The severity of the NBD can be thus classified as minor (0 to 9); moderate (10 to 20); or severe (21 to 41). It includes a scale from 0 to 10 to evaluate how satisfied an individual is with their bowel function, bringing into consideration a subjective perspective that complements the objective clinical analysis.

This tool adapts the NBDS created by Krogh et al. in 2009^14^, in order to address nuances that are specific to pediatric NBD, which can influence its functional impact and family dynamics.

### Translation and back-translation

The questionnaire was translated by two bilingual Brazilian translators, one of whom was a health professional, while the other was not, ensuring its conceptual equivalence and cultural adequacy. These independent translations sought to produce a conceptual translation rather than a literal one, focusing on preserving the meaning of the questions.

Then, two pediatric NBD specialists analyzed the discrepancies in the two texts produced and consolidated them into a unified version, which was back-translated independently by two other translators, both native English speakers and fluent in Brazilian Portuguese. These stages ensured that the content was reliable and semantically clear. Finally, two specialists approved a final version of the questionnaire in Brazilian Portuguese.

### Cultural adaptation and semantic assessment

Using the Delphi method^25^, a committee of five professionals specialized in child health and NBD, all of whom were at least Masters, was formed to analyze the preliminary version. After agreeing to participate by signing an informed consent, they were sent a Google Forms link containing the preliminary version of the survey.

Each question was analyzed for its conceptual, linguistic, semantic, and experiential equivalence using a five-point Likert scale. The conceptual equivalence was analyzed to ensure that the meaning was in line with the original goal; the linguistic equivalence, to adjust idiomatic and colloquial expressions that could not be translated literally; the semantic equivalence, to ensure that the original meaning of the words was maintained; and the experiential equivalence, to align the terms with the cultural experience of the target population^25^.

Adjustments were made following suggestions from the specialists in all questions whose mean clarity was different from 5 points on the Likert scale. Kendall’s Coefficient of Concordance (W)^27^ was used to evaluate the inter-evaluator consistency. Questions with W ≥0.8 were approved for the final version of the Brazilian Portuguese survey. This process ensured the necessary equivalence to validate the applicability and reproducibility of the instrument in the Brazilian context.

### Pretest

The NBDS was applied to two groups of pediatric patients being monitored in a children’s rehabilitation center. In group one, we included five individuals who used TAI as an NBD management strategy. Group 2, in turn, included five who used conservative measures other than TAI. They evaluated whether the survey was clear and how easy the questions were to understand, scoring them on a Likert scale.

Clinical-demographic variables, such as sex, age, ethnicity, family income, clinical diagnosis, functional classification, and mobility were collected. The pediatric NBDS and complementary indices, such as Wexner’s Score, also known as the Fecal Incontinence Severity Scoring System, were used to evaluate differences between the groups.

They were applied by a pediatrician with over 10 years of experience in the field of intestinal rehabilitation at the SARAH Network of Rehabilitation Hospitals, working directly with children and adolescents with NBD, who analyzed whether the instrument was applicable and well-adapted to the Brazilian context, identifying the necessary adjustments to optimize its use.

### Statistical analysis

A Likert scale was used to evaluate how easy the questionnaire was to understand and its equivalences. The consistency among committee experts was evaluated using Kendall’s Coefficient of Concordance (W)^27^, where W ≥0.66 indicated a high concordance level, and W ≥0.8 meaning that it was approved.

In the pretest analysis, categorical variables were described in percentages, while continuous variables were described using means and their respective standard deviations (SD). To compare the groups, Fisher’s exact was applied to categorical variables, while continuous ones were analyzed using Student’s *t* depending on data distribution, which was previously assessed using the Shapiro-Wilk test.

The level of statistical significance considered was *P*<0.05. Analyses were conducted in the software IBM SPSS Statistics for Windows, version 29, (2023) and Graphpad Prism, version 9, (2020), ensuring methodological rigor and statistical accuracy.

## RESULTS

**FIGURE 1:**
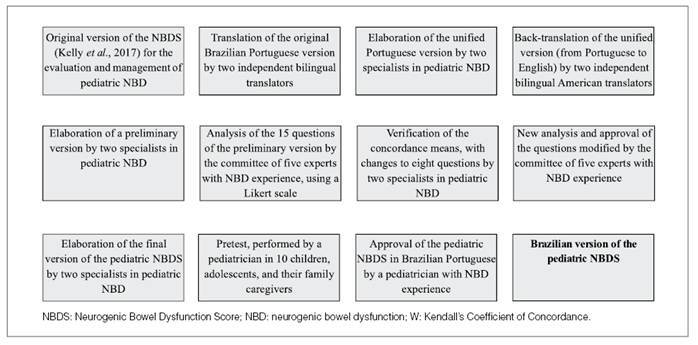
Flowchart of the steps of the translation of the Brazilian pediatric NBDS questionnaire, its cultural adaptation, and its semantic validation using a pilot study, Brazil, 2025.

The process of translation and cultural adaptation of the pediatric NBDS survey led to the creation of a Brazilian version that was equivalent to its original counterpart in its conceptual, linguistic, semantic, and experiential aspects (APPENDIX). Figure 1 shows a detailed flowchart of the steps necessary to produce a final version of the pediatric NBDS survey in Brazilian Portuguese.

**APPENDIX.**
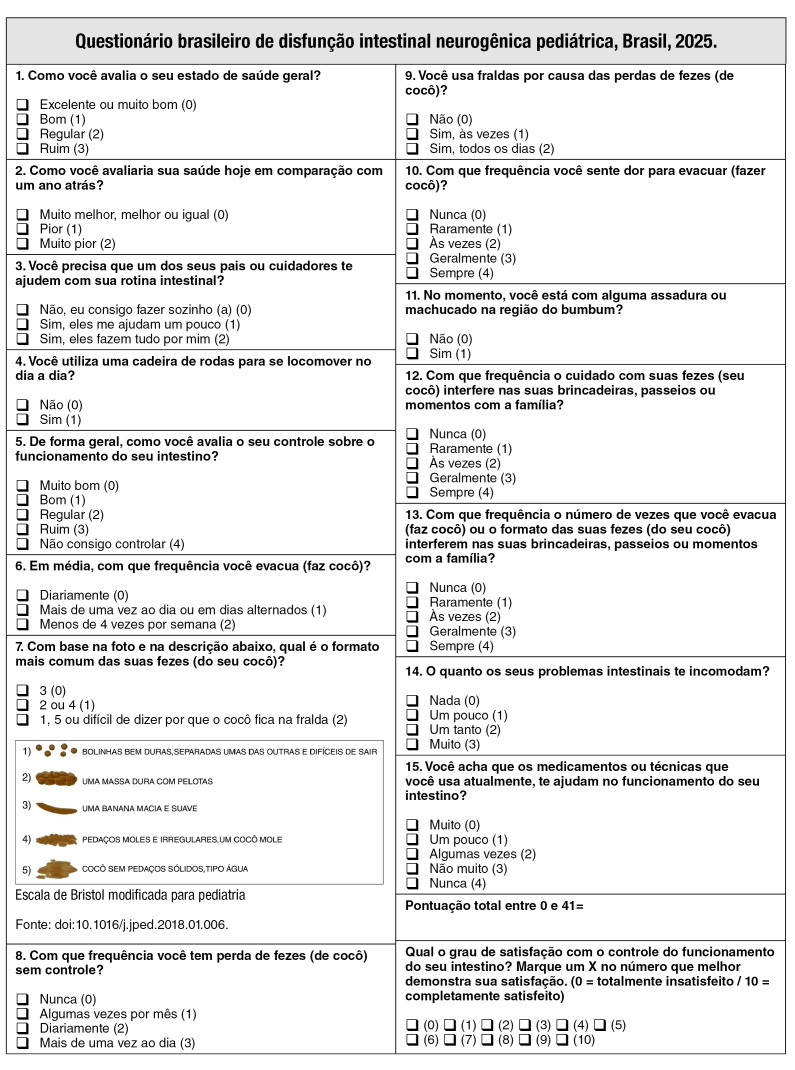
Brazilian version of the Pediatric Neurogenic Bowel Dysfunction Score (NBDS).

### Translation, back-translation and semantic validation

During the translation and back-translation steps, the 15 questions in the survey were analyzed by two experts in pediatric NBD, who made changes to the instrument to ensure that it was clear and appropriate to the Brazilian context. These adjustments aimed at adapting the questionnaire to the level of understanding of younger children, as well as caregivers and populations from different Brazilian socioeconomic and cultural contexts.

The expert committee evaluated the equivalence and clarity of the translation using a Likert scale, and the absolute mean scores that resulted are presented in Table 1.

**TABLE 1:** Evaluation of the equivalence and clarity of the 15 questions by the expert committee, Brazil, 2025.

Questions	Equivalence Assessment	Mean Equivalence Likert Scores	Mean Clarity Likert Scores
1, 2, 4, 6, 10, 13, 15	Conceptual	5	5
	Semantic	5	
	Linguistic	5	
	Experiential	5	
3, 12, 14	Conceptual	3	3
	Semantic	3	
	Linguistic	4	
	Experiential	3	
5, 8, 9	Conceptual	4	4
	Semantic	4	
	Linguistic	4	
	Experiential	4	
7	Conceptual	4	4
	Semantic	3	
	Linguistic	4	
	Experiential	4	
11	Conceptual	4	4
	Semantic	3	
	Linguistic	4	
	Experiential	3	

Likert mean: absolute mean on the Likert Scale: 1: very low, 2: low, 3: medium, 4: high, 5: very high.

Certain questions in the preliminary version of the pediatric NBDS survey were reviewed and changed, as Table 2 shows, according to issues identified by the expert committee, which included the simplification of technical terms, adaptation of idiomatic expressions, and the rewriting of questions that were too long or ambiguous.

**TABLE 2:** Modifications to eight questions from the preliminary version of the pediatric NBDS in Brazilian Portuguese and the mean degree of specialist concordance before and after adjustments, Brazil, 2025.

Q	Original content	Issue identified in the preliminary version	Modification Implemented	Mean W (Before)	Mean W (After)
**3**	***Does a parent or caregiver have to assist you with your regular bowel routine?***	The term “**regular bowel routine**” was considered too technical and not easily understood for lay caregivers and young children.	Simplified to “rotina intestinal”	0.72	0.85
**5**	***How controlled do you think your bowels are?***	The expression “**bowel control**” raised questions about what, exactly, would be evaluated as “control”.	Reworded to “**controle sobre o funcionamento do seu intestino**”	0.69	0.82
**7**	***Using the attached descriptions on average what category was your poop most commonly?***	“**Category**” was considered a technical term.	Reworded to “**formato mais comum das suas fezes**”	0.73	0.84
**8**	***How often do you have stool accidents?***	“**Stool accidents**” was seen as a confusing expression, that would not be familiar to lay caregivers and smaller children.	Reworded to “**perda de fezes sem controle**”	0.70	0.83
**9**	***Do you wear a diaper for stool accidents?***	Repeats the term “**stool accidents**”.	Replaced with “**por causa das perdas de fezes**”	0.68	0.82
**11**	***Do you currently have any rashes or cuts on your bottom?***	The term used in the translation was the more technical equivalent of “anal fissure”, which was found to be little familiar to lay caregivers and smaller children.	Replaced with “**machucado na região do bumbum**”	0.73	0.85
**12**	***How often does the management of your stool interfere with your recreational, social or family activities?***	The preliminary version used the term ‘bowel management,’ which was unfamiliar to lay caregivers and young children.	Reworded to “**o cuidado com suas fezes**”	0.71	0.84
**14**	***How bothersome are your bowel problems to you?***	“**Bothersome**” was considered too subjective.	Replaced with “**o quanto os seus problemas intestinais te incomodam**”	0.72	0.85

Mean W before and after: mean Kendall’s coefficient of concordance among specialists before and after adjustments; Q: question.

After these adjustments, all questions reached a very high equivalence and clarity in the Likert scale, and Kendall’s Coefficient of Concordance (W) reached values above 0.8, indicating a high agreement among specialists, as Table 2 shows.

### Clinical-demographic characterization of pretest groups

The groups were homogeneous in regard to the variables analyzed, with the exception of sex (*P*-value=0.024). Most participants in Group 1 (TAI users) were male (80%), while Group 2 (non-TAI users) included only girls. The other clinical-demographic variables, including age, clinical diagnoses, family income, and functionality showed no significant differences, as Table 3 describes.

**TABLE 3:** Clinical and demographic characterization of the pretest groups, Brazil, 2025 (n=10).

	GROUP 1 (n=5)	GROUP 2 (n=5)	**Fisher’s Exact Test and Mann-Whitney U Test (*P*-value)**
Sex	80% Male	100% Female	0.024
	20% Female		
Mean age in months (SD)	148 (±27.7)	113 (±18.1)	0.056
Ethnicity	60% White	20% White	0.167
	20% Black	40% Black	
	20% Brown	40% Brown	
Degree of kinship of the family caregiver	80% Mother	80% Mother	0.278
	20% Father	20% Aunt	
Monthly income in minimum wages	20% up to and including 1	40% up to and including 2	0.171
	40% up to and including 3	20% up to and including 3	
	20% up to and including 5	20% up to and including 5	
	20% above and including 7	20% above and including 7	
Diagnoses	100% Myelomeningocele	100% Myelomeningocele	N/A
	100% NBD	100% NBD	
	100% NB	100% NB	
Functional classification	20% Sacral	40% Low Lumbar	0.083
	60% Low Lumbar	20% High Lumbar	
	20% High Lumbar	40% Thoracolumbar	
Mobility	80% Community Walker	20% Community walker	0.079
	20% Non-Walker	20% Restricted walker	
		60% Non-Walker	
CIBL (Self-Cat)	100% Yes (40% self-cat)	100% Yes	0.222
Urinary incontinence	80% Yes	40% Yes	0.238
	20% No	60% No	
VPS	60% Yes	40% Yes	0.397
	40% No	60% No	
Third ventricle ostomy	100% No	20% Yes	0.500
		80% No	

SD: standard deviation; NBD: neurogenic bowel dysfunction; NB: neurogenic bladder; CIBL: clean intermittent bladder catheterization; Self-Cat: bladder self-catheterization; VPS: ventriculoperitoneal shunt.

### Clarity and ease of understanding of the questionnaire

The clarity and ease of understanding of the questions were evaluated using a Likert scale. The mean score was 4.8 (±0.1) in Group 1 and 4.6 (±0.1) in Group 2. Both groups considered the questions to be clear and understandable, with no significant differences between them (Table 4).

**TABLE 4:** Comparison of results between pretest groups, Brazil, 2025 (n=10).

		GROUP 1 (n=5)	GROUP 2 (n=5)	Gamma*	*P*
**Clarity and comprehension ratings of pediatric NBDS questions (Likert Scale)**	1 = Very low	0	0	-0.3	0.513
	2 = Low	0	0		
	3 = Medium	0	0		
	4 = High	1	2		
	5 = Very high	4	3		
**Pediatric NBDS Score**	0 to 9 = Mild NBD	1	0	1	**0.004**
	10 to 20 = Moderate NBD	4	1		
	21 to 41 = Severe NBD	0	4		
**Satisfaction score for bowel function control**	0	0	2	-1	**0.008**
	1	0	0		
	2	0	1		
	3	0	1		
	4	0	0		
	5	0	1		
	6	0	0		
	7	0	0		
	8	1	0		
	9	1	0		
	10	3	0		
**Wexner Score**	1 to 7 = Mild FI	5	0		
	8 to 15= Moderate FI	0	2	1	**0.008**
	15 to 20 = Severe FI	0	3		

*Gamma: Goodman-Kruskal gamma correlation; NBDS: Neurogenic Bowel Dysfunction Score; FI: fecal incontinence.

### Pediatric NBDS total scores and satisfaction with bowel function control

The total score of the pediatric NBDS was significantly different between the groups, with means of 13.2 (±2.4) in Group 1 and 23.6 (±3.5) in Group 2 (*P*-value=0.008). These results suggest that the group that used TAI as a strategy to manage NBD presented a less severe cases of the disease (Table 4).

Furthermore, Group 1 showed significantly higher scores of satisfaction with bowel functioning. In a scale from 0 to 10, they had a mean of 9.4 (±0.9), while in Group 2, the mean was 2.0 (±2.1) (*P*-value=0.008).

### Severity of neurogenic bowel dysfunction

The classification of NBD severity showed that Group 2 had more serious cases, while in Group 1 there were more moderate ones, reiterating the positive impact of TAI in reducing the severity of NBD.

## DISCUSSION

This study translates the (NBDS) into Brazilian Portuguese, adapting it culturally and validating it, which significantly contributes to evaluate NBD in children and adolescents with SB. Our results suggest that the translated and adapted version is clear, understandable, and applicable to the Brazilian context, attending to the linguistic and cultural needs of the pediatric population.

The translation and cultural adaptation of instruments such as the NBDS are essential to ensure that linguistic and cultural nuances are preserved, and conceptual equivalence is maintained^24^. To achieve this, systematic methods such as the Delphi^25^ technique and back-translation were employed, ensuring fidelity to the original instrument and adherence to international recommendations for cross-cultural adaptation^17^ ^-^ ^19^ ^,^ ^24^ ^,^ ^26^. The original version of the questionnaire, provided by the manufacturer Coloplast GmbH, was also consulted to ensure content consistency^20^. These procedures align with international recommendations for cross-cultural adaptation of assessment tools, which emphasize conceptual, semantic, and operational equivalence to ensure validity across cultures^24^ ^,^ ^26^. Notably, previous studies on the adaptation of the NBDS into other languages did not detail the methodological stages of translation, back-translation, cultural adaptation, or semantic validation^17^ ^-^ ^19^, highlighting that the present study is the first to apply a comprehensive and rigorous adaptation protocol to the NBDS.

A Kendall’s Coefficient of Concordance of W >0.8, reached after adjustments, showed that the experts had a consistent opinion, giving credence to the fact that this survey is adequate for its target audience^27^.

The clinical and demographic evaluation of the pretest showed significant differences in the outcomes between groups, especially regarding the severity of the NBD and the satisfaction with bowel functioning control. These results are in line with previous studies^1^ ^,^ ^23^ and Kelly et al.^9^, who showed how efficient transanal irrigation (TAI) is in reducing the severity of NBD symptoms^9^ ^,^ ^21^ ^,^ ^22^. Group 1, which used TAI, had a significantly lower score on the NBDS, indicating that their NBD was less severe and the individuals in the group were more satisfied with their capacity to control their bowel function.

Although both groups were homogeneous for most demographic variables, there was a significant difference in sex (mostly males in Group 1, only females in Group 2). Previous studies suggest that sex and age may influence adherence to NBD management strategies and quality of life perception^7^ ^,^ ^13^. Nevertheless, further research is necessary to understand whether these differences could impact these results in the long term.

The results found also highlight the psychosocial impact of NBD, often neglected in clinical practice. Verhoef et al.^7^ emphasized that NBD compromises physical health, self-esteem, school performance, and social integration. The use of validated instruments, such as the pediatric NBDS, enables a more comprehensive and objective assessment of the impact NBD can have in these areas, enabling the elaboration of more effective and better targeted interventions.

Finally, the implementation of an instrument validated for Brazilian Portuguese fills an important gap in national clinical practice, seen as other countries have shown how the NBDS helped plan individualized therapeutic strategies and monitor interventions^15^ ^-^ ^19^. In Brazil, similar efforts have been made to culturally adapt gastrointestinal tools for the pediatric population, such as the Modified Bristol Stool Form Scale^28^, further highlighting the importance of contextually appropriate instruments to improve care and research. Moreover, standardization makes global comparisons possible, which in turn allows the elaboration of more effective public policies, especially in countries where the resources to manage NBD are limited^13^ ^,^ ^21^ ^-^ ^23^.

The questionnaire will be made publicly accessible as an open-access tool, ensuring unrestricted and free availability to broadly benefit the Brazilian patient population, while promoting its practical application and facilitating future research in the field.

### Study limitations and future direction

The small pretest sample restricted the generalization of our results. The demographic composition of the groups may also have influenced some of the differences observed. Future research, with larger and more heterogeneous samples, is needed to confirm our findings and explore how applicable the pediatric NBDS is in other subgroups of the pediatric population with NBD. Furthermore, we encourage the use of NBDS in different Brazilian clinical contexts, so its applicability can be evaluated in different settings.

## CONCLUSION

This is the first validation of the pediatric NBDS in Brazilian Portuguese, a significant advance for the integration of this instrument into the national clinical context. The translated survey was found to be clear, reliable, and sensitive, allowing for standardized evaluations of NBD in children and adolescents with SB. Its implementation contributes to standardizing clinical management using individualized approaches that are based on the severity of one’s condition. It also makes it possible to carry out global comparisons between different pediatric populations.

The translated and adapted pediatric NBDS is a robust tool that supports both clinical practice and research, helping define therapeutic interventions and longitudinal follow-up. Its application has the potential to improve the quality of life of children and adolescents with NBD and their families, promoting a comprehensive approach that considers both physical and psychosocial aspects of this condition. Furthermore, this tool can support the development of public policies for the specialized care of this population, helping improve the health system and increase the equity in the access to treatments.
